# Life on pause: An analysis of UK fertility patients’ coping mechanisms after the cancellation of fertility treatment due to COVID-19

**DOI:** 10.1177/1359105321999711

**Published:** 2021-03-08

**Authors:** Anna Tippett

**Affiliations:** University of Hertfordshire, UK

**Keywords:** COVID-19, emotions, infertility, in vitro fertilisation, mental health, wellbeing

## Abstract

In March 2020, fertility clinics across the UK began cancelling all assisted reproductive technology (ART) treatment, with the Human Fertilisation and Embryology Authority (HFEA) stopping all ART treatment from going ahead beyond the 15th April 2020 due to the COVID-19 pandemic. This article examines the coping mechanisms adopted by fertility patients during this time, focussing on the emotional support received from online fertility forums and fertility clinics during the indeterminate wait for treatment to resume. The study draws upon an online survey which assessed the mental health and wellbeing of 124 female fertility patients whose ART treatment was cancelled due to the Coronavirus pandemic. The findings indicate a potential for improved communication between fertility clinics and patients in order to reduce psychological stress and isolation during the postponement of ART treatment, alongside better utilisation of online platforms as mechanisms for support. This article adds to the growing body of knowledge concerned with the implications of denying reproductive rights to the infertility community during a global pandemic. It also contributes to sociological discussions on the support mechanisms available to those navigating infertility and the wider social management of uncertainty.

*‘I feel lost. . .I’m just waiting for treatment to begin’.*

The cancellation of assisted reproductive technology (ART) treatment in the UK from the end of March 2020 to mid-May 2020 due to the COVID-19 pandemic presented fertility patients with an emotional predicament no self-help book had prepared them for. Media headlines at the time aptly summarised the emotionally exhaustive state many women and couples had found themselves in: ‘*“Time is precious in IVF”: the women who fear they have lost their chance to have children*’ (Kale, 2020); ‘*All IVF treatment cancelled in “most difficult decision” watchdog has ever made*’ (Donnelly, 2020); ‘*Lockdown makes us fear we’ll never be parents: Couples whose IVF has been put on hold reveal their heartache’* (Webber, 2020). The mood within online infertility forums during this time was one of despair and sorrow. With no clear timeline to interrogate, it was unclear as to whether the ban on fertility treatments would go on for weeks or months.

The coping mechanisms adopted during this period of uncertainty presented a pertinent sociological and psychological landscape to explore. As the uptake of in vitro fertilisation (IVF) treatment has steadily increased since the early 1990’s, with more than 54,000 patients undergoing around 75,000 fertility treatments in the UK in 2017 (HFEA, 2017) and more than 250,000 babies being born as a result of IVF in the UK since 2016 (Press Association, 2016), it was clear that the ramifications of cancelled treatment would impact upon many women, couples and their wider families. This article contributes to the wider pool of sociological research into fertility treatment, providing an analysis of an unprecedented time in the infertility community through examining the following research questions: How was the mental health and wellbeing of female fertility patients affected after the cancellation of their fertility treatment?; To what extent did digital infertility communities help throughout this time?; and, how effective was the communication and support delivered by fertility clinics during this period of uncertainty?

This study draws upon an online questionnaire distributed to fertility patients in the UK whose fertility treatment was cancelled due to the COVID-19 pandemic. Although 129 responses were received, the majority of these (*n* = 124, 96%) were from female fertility patients, with this then constituting the focus of the study. The principal aim of this research is to contribute towards wider discussions on the mental health and wellbeing of women in the infertility community during the COVID-19 pandemic and UK lockdown. As successive waves of Coronavirus occur, or if another pandemic emerges entirely at a separate point in time, it is important that we learn from the experiences of fertility patients now in order to implement more streamlined measures of support in the future.

Existing studies have already drawn attention to mental health as an unavoidable component of undergoing fertility treatment (see Hernon et al., 2016; Hi-Kwan and Yuen Loke, 2015; Raguz et al., 2014; Shlomo, 2016; Skvirsky and Taubman-Ben-Ari, 2019). Indeed, Smeenk et al. (2004: 277) stress that patient’s mental health needs should be ‘considered an integral component of fertility care’, with Pasch et al. (2016) recommending that more attention needs to be given to the mental health needs of fertility patients and their partners. The emotional consequences of infertility are vast and include depression, anxiety, identity problems, loss of control, stigmatisation and fractured relationships (see Cousineau and Domar, 2007; Domar and Seibel, 1990; Lukse and Vacc, 1999; Moura-Ramos et al., 2016; Peterson, 2007), particularly amongst couples who have a long history of infertility and have experienced treatment failure (Chiaffarino et al., 2011). Hasanpoor-Azghdy et al. (2014) analysed the emotional-psychological consequences of infertility among infertile women seeking treatment and deduced a range of cognitive and emotional-affective reactions to infertility and the therapy process. These ranged from reduced self-esteem, feelings of failure, anxiety, worry, depression and hopelessness. Gdańska et al. (2017) have drawn attention to the prominence of stress induced by infertility, which can in turn ‘negatively affect the outcomes of infertility treatment’. Furthermore, research by Gameiro et al. (2014: 2239) has highlighted the importance of the role of fertility staff in preparing patients for the ‘possibility of treatment failure’ and the ‘associated grief process’, alongside the ‘positive effect of refocusing their life goals’.

Yu et al. (2014) have conversely identified a positive psychological response to the struggle of infertility. By applying Tedeschi et al.’s (1998) concept of ‘posttraumatic growth’ (the growth experienced by individuals arising from traumatic events) to the infertility community, they associated resilience and social support with enhanced levels of posttraumatic growth amongst women with infertility. Such social support is increasingly being sought via online infertility forums. Infertility research has focused on the internet as a tool to reduce feelings of isolation amongst those in the trying to conceive (TTC) community. Such research has pointed to ‘communities of support that supplement the real world’, with clinicians being encouraged to direct patients to such forums (Hinton et al., 2010: 440) and more web-based resources being encouraged to meet patients’ needs (Brochu et al., 2019). As a consequence of this, those within the infertility community are turning to the internet to extend their coping mechanisms. Research by Lundin and Elmerstig (2015: 444) examined internet support groups with a focus on involuntary childlessness and found that online forums provided emotional coping support ‘through the mutually supportive tips, advice and information shared by the participants’.

The social support offered by online infertility forums at the time of ART treatment cancellation in the UK due to COVID-19 offered a pertinent landscape to explore emotional responses and coping mechanisms within the TTC community. The emotional effects of infertility in the context of a global pandemic amplified what existing research has already revealed. As stated by Trinchant et al. (2020: 152), ‘reproductive rights are human rights’ and the negation of such rights for those facing infertility during the time of the initial COVID-19 outbreak served to increase emotional distress and anxiety, especially for those with longer infertility history. Research has shown that approximately two thirds of patients left waiting for ART treatment to resume expressed the will to proceed with their treatment during the pandemic (Esposito et al., 2020), with 50% of respondents in another study reporting clinically significant depressive symptoms due to suspension of their treatment (Gordon and Balsom, 2020). Patients were not only dealing with a sense of powerlessness but were also anxious at the thought of further compromising their chances of pregnancy due to deterioration of egg reserve and quality, particularly for older individuals/couples (do Carmo Borges de Souza et al., 2020). This article adds to the existing research on the mental health of fertility patients during this time and considers the utility of online infertility forums as platforms for additional social support.

## Methods and data

There is limited research to date which has considered the cancellation of fertility treatment due to factors outside of the patient’s individual medical circumstances. This study therefore explores the emotional experiences of female fertility patients during the UK lockdown in order to better understand the impact of treatment cancellation on mental health and wellbeing. An online survey was distributed via several online infertility forums throughout the month of May 2020. This month saw the government announce fertility clinics’ right to apply to reopen from the 11th May and thus provided a fitting window to explore how female fertility patients had coped during the waiting period for their clinic to reopen. Private Facebook groups with key words including ‘IVF’, ‘IUI’, ‘Infertility’, ‘Fertility’ and ‘Support’, constituted the main platforms where the survey was shared. It was also shared on the Fertility Network UK forum and the infertility support forums on Mumsnet and Netmums. The anonymity of the survey ensured the collation of honest and authentic responses, giving women the opportunity to contribute their own experiences to wider ‘theoretical categories’ (Vainio, 2012: 690).

The survey consisted of both open and closed questions, offering respondents a platform to elaborate on some of their answers. While closed questions offer a more quantifiable means of data analysis, enabling clear trends and generalisations to be made, open questions can add a richness to survey results (Krosnick, 2018) that cannot be reached by closed questions alone. The data collection tool used was Jisc Online Surveys which allowed for a fast, efficient and COVID-secure way to collect the data, whilst also ensuring anonymity for participants. The development tools offered by Jisc Online Surveys ensured clear signposting throughout the survey, enabling participants to complete the survey themselves. Although the initial target population consisted of fertility patients and their partners (where applicable), the call to participate was overwhelmingly answered by female fertility patients (*n* = 124, 96%). Due to this, the analysis was streamlined to focus solely on female fertility patients, with five partners of fertility patients (three females: two males, constituting 4% of the survey population) being eliminated from the study. The lack of available data on how partners of fertility patients coped during this time is thus an area that warrants greater attention. Most participants (90%) identified as heterosexual, with 8% identifying as gay/lesbian and 2% identifying as bisexual. In terms of age, 86% of respondents were in the 26–39 bracket, with 5% being in the 18–25 age bracket and 9% being in the 40–50 age bracket. About 82% (*n* = 102) of the sample did not have children, with 18% (*n* = 22) already having one child or more.

Several precautions were taken to uphold the trustworthiness of the study as the ‘validity, credibility, and believability of the research’ (Harrison et al., 2001: 324) are of utmost importance to social scientific investigation. The first precaution taken was the adoption of an online survey which incorporated both quantitative and qualitative questions. As this method is widely used within the social sciences, it offered a well-established and thus reliable platform to collect data. Shenton (2004: 64) has emphasised that ‘methods of data analysis, should be derived, where possible, from those that have been successfully utilised in previous comparable projects’. The prevalence of online surveys in social research, alongside the wider issue of having to adopt a COVID-safe methodology, ensured a well-tested methodology was being utilised. The second precaution taken was to ensure the safety and security of respondents. The anonymity of respondents, alongside the knowledge that they could withdraw from the study at any time without disclosing an explanation, created a safe environment for them to freely share their feelings and experiences. It is widely acknowledged that, in survey research, ‘issues of confidentiality and anonymity are central’ (Maruyama and Ryan, 2018: 77). A further precaution taken was to ensure support networks were available to respondents should they need them. This was done by sharing contact details of myself as the researcher, alongside the contact details of Fertility Network UK, a charity who run online support groups and webinars and have a support and information phoneline.

The qualitative data generated from the survey allowed for an inductive thematic analysis to be implemented, where dominant themes emerged from the data. This approach is rooted in grounded theory which ‘emphasises the technique of staying open’, enabling the researcher to ‘generate findings and themes directly from the data’ (Liang, 2013: 53). Although the survey was designed with a deductive method in mind, where principal frameworks were formulated according to the aims and scope of the research, the dominant themes within each framework were generated according to Braun and Clarke’s (2006) six-step process: (1) familiarising yourself with the data, (2) generating initial codes, (3) searching for themes, (4) reviewing themes, (5) defining and naming themes and (6) producing a report/discussion of the themes. Braun herself has emphasised the importance of describing the engagement with each of these stages in a ‘reflexive, contextually located way’ (Braun et al., 2019: 9). Table 1 consequently outlines how the six steps were approached in this study.

**Table 1.** Reflecting on the six-step process.

Step taken	Reflective account
(1) Familiarising yourself with the data	The data was analysed once the survey was taken offline. Both quantitative and qualitative responses were closely examined in order to get an overall picture of what the takeaway messages from the data were.
(2) Generating initial codes	Upon extensive analysis of the data, codes were formulated which reflected the main features of the responses. As ‘codes are the smallest units of analysis that capture interesting features of the data’ and serve as ‘building blocks for themes’ (Clarke and Braun, 2017: 297), formulating codes was a crucial part of developing overarching themes.
(3) Searching for themes	The data was repeatedly examined in order to establish recurring themes, built from the clustering of initial codes. Analytical observation and the frequency of the codes generated helped establish the themes.
(4) Reviewing themes	Clarke and Braun (2017: 297) state that ‘themes provide a framework for organising and reporting the researcher’s analytic observations.’ Through identifying and interpreting key features of the data guided by the wider research questions, themes were reviewed in-line with the wider objectives of the research.
(5) Defining and naming themes	The themes identified reflect the ‘interpretive depth’ (Braun and Clarke, 2014: 26152) of the data analysis process. The themes were named according to the collective meaning of the codes that sat beneath them. The initial two themes (*1. Psychological Stress and 2. Emotional Health and Wellbeing*) tour the first research question of this project which interrogates the mental health and wellbeing of female fertility patients. The second two themes (*3. Isolation and Lack of Support or Communication and 4. Positive Support Mechanisms*) represent the strengths and weaknesses of the communication and support received during treatment cancellation, thus addressing the remaining research questions in this project.
(6) Producing a report/discussion of the themes	The discussion of the themes is laid out in the sections that follow. The intention was to structure this article so that the codes sat beneath the themes in the discussion. You will therefore see both the overarching themes and codes represented in each section of the discussion.

Following on from this overview of the research model and data analysis, Table 2 gives an overview of the codes identified and the overarching themes they correspond to.

**Table 2.** Codes and corresponding themes.

Codes	Themes
• Unable to cope • Additional stress • Negative impact of social media	Psychological stress
• Overwhelmed with emotion • Wanting others to understand • Disclosed cancellation of treatment to others	Emotional health and wellbeing
• Lack of support or communication from clinic/family/friends • Feeling alone/lost • Deficiency in understanding from others	Isolation and lack of support or communication
• Support from family/friends • Digital community support/communication • Clinic support/communication	Positive support mechanisms

The following discussion provides a qualitative analysis of the participants’ answers, focusing on the central themes identified in the above table.

## The impact of treatment cancellation on stress levels

The prevalence of stress amongst those surveyed occurred frequently in the analysis. Three dominant codes emerged from the data: (1) weakened coping mechanisms, (2) additional stress and (3) the negative impact of social media during this time. Stress in this context refers to heightened feelings of depression, anger or anxiety as a result of both treatment cancellation and the surrounding impact of the UK lockdown. With access to support being online-only, and social media reminding fertility patients of what many of them did not have, stress levels increased across a large proportion of the sample. Indeed, the delays and uncertainties experienced due to fertility clinic closures have been linked to anxiety, depression and immense stress (Bigg, 2020).

### Weakened coping mechanisms post-treatment cancellation

Several respondents expressed difficulty in managing treatment cancellation, with some associating their inability to cope with a lack of communication received from their clinic.

**Table:** 

‘I have felt unable to cope. I felt like I was drowning in my own grief. The uncertainty has been killing me since I’m nearly 40’ (26–39, heterosexual)
‘The updates were not regular enough and vague. I think that, if this is repeated with another closure, people won’t cope very well. It is too stressful sadly’ (40–50, heterosexual)
‘I have withdrawn from my partner and haven’t really accepted the support. Not good as it has put more strain on mine and my husband’s relationship’ (26–39, heterosexual)
‘I feel negative, down and bitter towards the fact that I’m just waiting around and there’s nothing I can do to take steps towards starting treatment’ (26–39, heterosexual)

Although patience is a prerequisite of the TTC journey, the uncertainty of when treatment would be allowed to resume presented patients with an unparalleled circumstance in which they were stripped of all power and choice. This notion of powerlessness coincides with Pitts (2005: 244) concept of the ‘fixed body’ which considers the body as underprivileged and thus unworthy of the postmodern flexibility and choice of capitalist-driven consumption. The fixed body in this context is characterised by stagnancy; an undetermined period of waiting which, for some, allowed for further deterioration of egg numbers and quality due to age. Because of this, respondents were not able to cope in ways they had done previously whilst being in-between cycles or waiting for communication from their clinic. When asked to rate how they had been coping emotionally during treatment cancellation on a scale of 1–10, with 1 being ‘not coping well’ and 10 being ‘coping extremely well’, 61% (*n* = 76) of the sample selected 5 or below, with 9% (*n* = 11) of the sample selecting 9 or 10.

### Psychological stress

Respondents described how the cancellation of treatment had added another layer of stress to an already exhaustive emotional journey.

**Table:** 

‘It’s been very difficult; I’ve had sleeping issues and been very stressed’ (26–39, heterosexual)
‘[My treatment] has been cancelled twice during the Covid-19 outbreak. I now have to delay treatment due to work commitments. No support can really help with that’ (26–39, bisexual)
‘It has been additional stress to contend with during the lockdown’ (26–39, gay/lesbian)
‘I am very hurt and angry about the HFEA decision and how fertility patients have been treated, as well as how other vulnerable groups have been treated. The Government should be ashamed. The lives lost and the lives of the babies who will never be born. . .the Government needs to take responsibility and apologise to the nation’ (26–39, heterosexual)
‘Stress of keeping it secret, two of my friends have got pregnant/had babies in lockdown’ (26–39, heterosexual)

The increased levels of stress described by respondents highlights the impact treatment cancellation had on their lives. Stress, anxiety and coping mechanisms have been frequent themes examined in infertility research, particularly in terms of outcome success (see Boivin and Takefman, 1995; Heredia et al., 2020; Maroufizadeh et al., 2019; Miller et al., 2019; Peterson et al., 2006; Rooney and Domar, 2016; Smeenk et al., 2005). It is thus questionable as to whether the heightened stress experienced by fertility patients during the UK lockdown led to increased levels of anxiety after treatment resumed. Analysis into treatment success from mid-May to December 2020 would be beneficial in terms of measuring the impact this psychological stress may have had on treatment uptake, experiences and outcomes.

The impact of psychological stress beyond the wait time alone is also worth noting. When clinics could apply to reopen in mid-May of 2020, precautionary social distancing measures were implemented, which included the wearing of masks by both staff and patients and, most significantly, the exclusion of spouses/partners from entering clinics to ensure a lower footfall. Although this survey did not include an analysis of these social distancing measures (as it was distributed just before the reopening of fertility clinics), analysis of fertility forums from late May onwards reveals heightened anxiety amongst patients at the thought of having to attend scans and procedures alone. Increased levels of stress and anxiety surrounding the exclusion of partners from the IVF process marked another significant period in ART treatment during COVID times and will require further interrogation in terms of the impact this had on relationships, wellbeing and the emotional inclusion of partners throughout the IVF/IUI journey.

### Negative impact of social media

The internet is both empowering and disempowering for those anticipating or undergoing fertility treatment. During the closure of fertility clinics, the internet became a complex tool for women whose treatment had been cancelled. Although fertility forums were largely considered a positive outlet (discussed in the final theme *Positive Support Mechanisms*), social media became a problematic form of communication during the UK lockdown, with patients feeling emotionally triggered by exposure to pregnancy or child-related content.

**Table:** 

‘Seeing so many pregnancy announcements [online] really upset me. My only thought of not seeing them any more would be to delete all social media’ (18–25, heterosexual)
‘I have seen lots of posts commenting on how easy the childless must have it during lockdown. I found these posts very distressing and upsetting’ (26–39, heterosexual)
‘Constant comments of the baby boom in 9 months from lockdown. Pregnancy announcements. People saying how unnecessary IVF is and a waste of NHS money – should adopt instead’ (26–39, heterosexual)
‘Social media just shows me people I know that have fallen pregnant so easily, accidents etc. It hurts as we have had the hardest journey I could ever imagine’ (26–39, heterosexual)
‘Sick of people complaining online about their kids in lockdown’ (26–39, gay/lesbian)
‘Everyone is struggling I understand that, but those who have children are able to spend so much time with them and all I keep seeing is everyone whining about their kids misbehaving! I’d give anything for a child, even if they were to misbehave’ (18–25, heterosexual)

About 51% (*n* = 63) of respondents felt that social media had both a positive *and* negative impact on their mental health whilst waiting for clinics to reopen. 32% (*n* = 40) of respondents either agreed or strongly agreed that social media had a *detrimental impact* on their mental health during this time, with 10% (*n* = 13) of the sample either agreeing or strongly agreeing that social media had a *positive impact* on the mental health during the waiting time. The remaining 7% were either undecided (5% *n* = 6) or did not use social media (2% *n* = 2).

The impact of social media on mental health and wellbeing during clinic closure was a significant finding in this analysis. Although the associated harms of social media are potentially unavoidable for those who engage with it, an opportunity for social media to be used as a platform of inclusivity and support was something missed by several clinics in the UK. About 59% (*n* = 68) of respondents who were a member of an online fertility forum stated that they engaged with the digital fertility community they were a part of more frequently since the cancellation of their treatment. This is an area that should be more readily utilised by clinics to improve the support measures available to their patients should a similar situation arise in the future.

## Emotional health and wellbeing

The theme of emotional health/wellbeing consistently occurred within the data. There was a focus on heightened emotional responses, the desire for others to understand, and the subsequent disclosure of treatment to others. This bears parallels to Van den Akkers et al. (2017) research which emphasised *concerns about disclosure* and *motives for disclosure* as important findings in their study into disclosing assisted conception treatment at work. With personal and professional boundaries being involuntarily merged throughout the entirety of the UK lockdown, fertility patients in employment were presented with a complex situation in which the demands of work commitments seeped into their homes. Boyle’s (2000) work on emotional processes at work offers fitting insight into this, as she draws attention to emotional culture, linking this to Goffman’s (1959) concept of ‘offstage spheres’; physical realms found outside of the workplace itself, such as family or household. Patients dealing with the cancellation of fertility treatment were also contending with the infringement of professional demands in their offstage environments which likely had further impact on their emotional health and wellbeing.

### Heightened emotional responses

The emotional strain experienced by respondents was evident in the qualitative data provided. The indeterminate waiting time was a recurrent challenge, alongside social media acting as a trigger for resurfacing emotions.

**Table:** 

‘The unknown of the future has been overwhelming to me’ (26–39, heterosexual)
‘The whole cancellation of the treatment has had a bad impact on my mental health. As a sufferer of depression and anxiety, this really was the last thing I needed. Each day has been a battle’ (18–25, heterosexual)
‘Absolute hell. IVF is hard and waiting for IVF is hard. Waiting to find out if my eggs are even viable. . .is unbearable. Feel isolated and hopeless. I’m literally in limbo and cannot stop crying’ (26–39, heterosexual)
‘The unknown is the worst. And the waiting. It’s like just putting your life on hold’ (26–39, heterosexual)
‘It’s impossible for anyone not going through infertility to understand. So, when people say, “oh it won’t be long until it starts again”, they are totally unaware of the emotional strain and psychological affect it has. It put us back a further 4 months and that’s after we have already been waiting 2 years’ (26–39, heterosexual)
‘The fact you have no idea what posts you’ll see until you open up social media it allows for a lot of shock and upset moments as you’re completely unprepared for a post that might trigger an emotional feeling you’re not expecting or wanting in that moment. You then start to feel overwhelmed by it all and pretty much have to not go on social media so you can avoid the upset that a random post might cause. That inadvertently makes you feel more alone as you’re then missing out on seeing friends lives etc. that you might have wanted to be involved in’ (26–39, heterosexual)

In terms of identifying the main sources of emotional support received, the most common category selected by respondents was spouse which was disclosed by 89% of respondents (*n* = 110). This was followed by friends (43% *n* = 53), online support forums/groups (43% *n* = 53), wider family (37% *n* = 46), pets (26% *n* = 32) and colleagues (17% *n* = 21). Emotional support received from fertility clinic counsellors was selected by only 5% (*n* = 6) of respondents, revealing a need for improved communication and support mechanisms in this area. Greater value must be placed on the emotional aspects of infertility within the sector as there appears to be an almost-exclusive focus on the medicalisation of the body which consequently serves to marginalise the importance of emotional health and wellbeing.

### Seeking understanding and disclosing treatment to others

Some respondents expressed an increased need to disclose their fertility treatment to people whom they may not have otherwise informed. About 24% (*n* = 30) of respondents stated that they had disclosed their treatment to more people since they learnt it would be cancelled.

**Table:** 

‘I wanted people to understand why my mood had deteriorated. I also felt better by talking about the fact that it had been cancelled’ (26–39, heterosexual)
‘Talking about it to anyone that would listen helped’ (26–39, heterosexual)
‘It was an additional stress added during the lockdown and I therefore gave in and told more people (only a couple more). I also had to inform my line manager as all the dates I had previously booked off as annual leave (to coincide with my cycle) needed to be cancelled and rearranged’ (26–39, gay/lesbian)
‘I am so upset and disappointed about waiting, I’ve felt I needed to explain why this is’ (26–39, heterosexual)
‘Although this cycle we decided to keep few people in the loop as possible, I am very open about receiving fertility treatment. When it was announced all fertility treatment would be frozen, I had a lot of friends message as they knew this would affect us. This is when I decided to share we had in fact just had egg collection’ (26–39, heterosexual)

The need to disclose treatment became particularly pertinent during the UK lockdown, with fertility patients wanting to justify the heightened stresses and emotions they were experiencing. Doing so allowed for more support mechanisms to emerge which upholds wider research recommendations surrounding the importance of psychological support before, during and after fertility treatment (see Heredia et al., 2020; Malina and Pooley, 2017; Zagami et al., 2019).

There was also hope that, through disclosure, greater understanding would be sought, not only on the part of understanding the emotional journey of the fertility patient, but also to encourage conformity to lockdown rules to ensure a more speedy return to normality and, consequently, to ensure the reopening of fertility clinics.

**Table:** 

‘Felt it was important for people to understand why they should follow the rules so we could get back to normal’ (26–39, heterosexual)
‘Was good to talk to others and explain the situation. I think it made friends and family understand why I was feeling quite upset and anxious’ (26–39, heterosexual)
‘There has been quite a lot of talk on social media about the hardships of those having to put off fertility treatment and I feel this has helped friends to understand more’ (26–39, heterosexual)
‘Seeing people disregard the lockdown rules made me so angry and I felt it was important for more people to understand the sacrifices people like me were having to make so that they would be more mindful of their behaviour’ (26–39, heterosexual)

## Feeling alone: Consequences of insufficient support and communication

The insufficient support and communication during the time of treatment cancellation constituted the most pertinent finding of this research. Respondents expressed feelings of isolation during the wait period, drawing attention to the problem of fertility clinics excluding emotional wellbeing from their services. From this perspective, if medical treatment is not actively happening, the fertility patient is left to deal with the psychological effects alone. Although some respondents disclosed that they were offered an online session with their clinic’s counsellor, this was sometimes at an extra cost. Research has indicated that rates of depression and anxiety are higher amongst women struggling with infertility and that couple relationships can also be negatively impacted (Bright et al., 2020). Better communication from clinics could thus have made a significant difference to the wellbeing of the fertility patients who were left waiting for treatment to resume.

### Lack of communication and support

Many respondents expressed disappointment with the lack of communication and support offered by their fertility clinics, with 69% (*n* = 86) stating they had been offered no support whatsoever from their fertility clinic after treatment cancellation. A number noted a complete absence of communication on the part of their clinic, with others revealing that they received one courtesy phone call, email or letter and nothing beyond this. About 41% (*n* = 51) of respondents either *disagreed* or *strongly disagreed* that they had received sufficient emotional support since the cancellation of their treatment. About 36% (*n* = 45) either *agreed* or *strongly agreed*, with the remaining 23% (*n* = 29) being *undecided*. Although emotional support comes in a range of forms, the notable lack of support offered by fertility clinics was apparent in the qualitative responses.

**Table:** 

‘No support other than my husband and cats. The fertility clinic’s message of ‘don’t ask us for timescales, don’t call us’ is really upsetting’ (26–39, heterosexual)
‘I have received no pro-active follow up from the clinic’ (26–39, heterosexual)
‘Didn’t receive one phone call from the clinic after our treatment got cancelled’ (26–39, gay/lesbian)
‘They [the clinic] sent a text. A week after the fact [of clinic closure] and I had still been injecting’ (26–39, heterosexual)
‘My clinic should’ve been updating their website and Facebook page. They haven’t. My clinic should’ve contacted every single patient. My clinic should’ve been more transparent. They’ve told many different patient’s different timescales for treatments. . .they’ve caused a lot of distress. It’s a private clinic too!’ (26–39, heterosexual)
‘I initially received nothing from my clinic and had to ring myself numerous times. . .the most ‘support’ offered has been small print on a letter reminding me of their counselling service’ (18–25, heterosexual)

Only 33% (*n* = 41) of patients had been contacted by their clinic again *after* they were informed of treatment cancellation, with the remaining 67% (*n* = 83) receiving no communication at all. Accessible psychological support, alongside more regular communication, would have improved the relationship between fertility clinics and their patients during this time. Indeed, research has shown that ‘infertility counselling offers the opportunity to explore, discover and clarify ways of living more satisfyingly and resourcefully when fertility impairments have been diagnosed’ (Van den Broeck et al., 2010: 422). The UK lockdown provided an additional emotional layer for fertility patients to have to contend with and could have been made less traumatic by simple check-ups over the phone.

**Table:** 

‘Routine contact, even if it was generic, would have made me feel less “forgotten”’ (40–50, heterosexual).
‘The fertility process needs far more compassion from staff delivering the service’ (26–39, gay/lesbian)
‘My clinic informed us of counselling, but I feel more check-up phone calls would have been nice’ (26–39, heterosexual).
‘Providing clearer communication by telling us if there was research being conducted about the virus and fertility. At this stage, we only know clinics will re-open, but it is still unclear whether the cycles can be conducted until the end, or whether it will be frozen cycles only’ (26–39, gay/lesbian).

Some of the responses implied that patients felt forgotten and that the simple inclusion of more phone calls could have provided them with much needed reassurance.

### Deficiency in understanding from others

Respondents expressed frustrations with the lack of understanding from family, friends and colleagues. In some cases, respondents also felt discriminated against because of their fertility impairments, highlighting unfavourable treatment when compared to couples who can conceive naturally.

**Table:** 

‘No one understands. Friends have just told me that it’s ‘for the best’ and not the right time to do IVF. Unless people have gone through it themselves, they just don’t get it’ (26–39, heterosexual)
‘People don’t understand and it’s genuine torture. I feel it’s discriminatory as fertile people are not being told to stop making babies, they are in fact joking about baby booms’ (26–39, heterosexual)
‘I am still struggling. Three close friends and colleagues have gotten pregnant within two months of trying. One the cycle before my IVF, one the cycle of and one the cycle after. Nothing has really helped as our hopes have been crushed and taken away, yet people are not stopped or discouraged from trying naturally if that is an option for them’ (26–39, heterosexual)
‘It has been very tough. I feel like no one understands how I feel so I keep it to myself’ (18–25, heterosexual)
‘Mostly, people don’t understand’ (26–39, heterosexual)
‘Family, friends and partner don’t understand. I’ve tried to seek help from like-minded women on online forums but they’re equally as traumatised by the suspension of fertility treatments’ (26–39, heterosexual)

The lack of understanding from others of the TTC journey was upsetting for patients during treatment cancellation. The insensitivity of what has been dubbed the ‘Coronababy boom’ was particularly challenging and mentioned by 10% (*n* = 12) of respondents.

**Table:** 

‘It has also been difficult to repeatedly see people joking about the ‘Coronababy boom’ during this time, and also those with children frequently telling those without children (i.e. me) that we have ‘no idea’ what lockdown really means if we’re going through it without children’ (26–39, heterosexual)
‘Seeing lots of posts about the ‘Covid Baby boom’ and how people are expecting lots of babies at the end of the year is difficult. It’s also difficult to see the memes circulating that are comparing parents to people who have no kids (telling us how lucky we are and to spare a thought for the people who can’t have a nap, go on a run etc. when they want). I’d swap it for a baby any day!!’ (26–39, heterosexual)
‘Nowhere seems to be sensitive to the issue. Lots of people and wider media make jokes about baby booms but comment negatively on fertility treatments’ (26–39, heterosexual)
‘Lots of posts about ‘lockdown baby boom’ and more time to see everyone else having babies/becoming pregnant are a little annoying if you can’t even get started on a proactive process such as IVF treatment’ (26–39, heterosexual)
‘People commenting negatively about fertility treatments starting again. Lack of understanding of people. Joke pregnancy announcements. Predicted baby boom. People spending more time that usual being inconsiderate’ (26–39, heterosexual)

Time became a precious commodity during the cancellation of fertility treatment, heightening levels of stress, anxiety and resentment amongst those left waiting. Indeed, time taken to achieve a live birth is essential to managing patients’ expectations during treatment (Sunkara et al., 2020), so any time lost is of grave detriment to the patient. The value of time in this context was discussed as not being understood by others; time to those not needing fertility treatment was deemed inconsequential and the ‘safer’ option.

## Positive support mechanisms

It was clear that respondents gained the most support from their spouses, followed by friends and wider family which aligns with expectations. However, the aim of this paper is to focus specifically on the support received from online support forums/groups, which 43% (*n* = 53) of respondents listed as a primary source of support, whilst also considering the support offered by fertility clinics. Only 5% (*n* = 6) of respondents listed their fertility clinic counsellor as a source of support and the reasons for this lack of uptake of fertility counselling need to be interrogated further. Positive interventions from fertility clinics will therefore be analysed in order to produce tangible recommendations for the future.

### Digital support from the TTC community

Digital support from the TTC community via online groups or forums was deemed particularly helpful by many respondents. About 89% (*n* = 110) of respondents considered themselves part of an online digital IVF/IUI community, with 59% (*n* = 65) of those respondents stating they engaged with their TTC community more frequently since treatment cancellation.

It was clear that social media usage overall increased during lockdown, with 40% (*n* = 50) of respondents stating their social media use had increased, and 36% (*n* = 45) saying it had remained about the same. Twelve percent (*n* = 15) stated that their social media usage had decreased during this time, with a further 10% (*n* = 12) stating their social media usage had fluctuated. Only 2% (*n* = 2) of respondents did not use social media. The use of technology during UK lockdown provided much needed social interaction and, for fertility patients, the opportunity to share their concerns and frustrations with others in the TTC community had a positive impact on their overall wellbeing.

**Table:** 

‘My very small circle around me have been great, they’ve tried to say the right things to be supportive, but I think mostly the online groups are more helpful as they can actually relate to you’ (26–39, heterosexual)
‘The online forums have been the best as it’s people going through the same thing, so you don’t feel so alone’ (26–39, heterosexual)
‘It was a relief to read forums about ladies/couples going through the same issues and feelings – did not feel so alone and knew if any advice was needed I could seek it in an environment where everyone else was in the same position as myself’ (26–39, gay/lesbian)
‘Support groups have given us people that understand our feelings’ (26–39, heterosexual)
‘The forums I follow are full of people in the same position. It makes it feel a lot less lonely’ (40–50, heterosexual)
‘The online forum helped me to share my frustration and fears. Also, to stay informed’ (26–39, heterosexual)

Seventy-six percent (*n* = 94) of respondents either agreed or strongly agreed that they often used social media to obtain information about the reopening of fertility clinics. Digital communities thus became sites of both information and support where patients could combat feelings of isolation and loneliness and come together to share experiences. Computer-mediated support has been found to be beneficial for those dealing with health-related stigma (Rains and Wright, 2016), with active participation in fertility forums being considered an important tool for coping with psychological stress (Gazit and Amichai-Hamburger, 2020). Online forums thus present fertility clinics and practitioners with communicative platforms that could be utilised more effectively to ensure more active dialogues between clinics and their patients should future clinic closures occur.

### Positive intervention from fertility clinics

Although many of the responses were critical of their fertility clinic’s service and aftercare, it should be noted that clinics were faced with an unprecedented and complex landscape to navigate during this time. With government advice constantly changing and large numbers of clinic staff being furloughed, communication efforts were problematic to implement and monitor.

The efforts of some fertility clinics were mentioned by a small number of respondents who noted that online support groups, phone calls, emails, online question and answer sessions, fertility yoga and the offer of counselling made a difference to their wellbeing during this time. Maintaining a line of communication with patients provided an extra layer of support during the lockdown that was received positively by those affected by such efforts.

**Table:** 

‘I’m part of a Facebook support group for our clinic and we have all been talking most days. Kept me thinking positive when I’ve been struggling to do that’ (18–25, heterosexual)
‘The IVF Support UK Group on Facebook has been invaluable when I’ve been at my lowest. . .my clinic has also got a Facebook group which has been supporting patients through this time’ (26–39, heterosexual)
‘The counselling did not help but I am still grateful the clinic thought to provide this service’ (26–39, heterosexual)
‘Our clinic has been excellent – they have kept in touch and enquired about our wellbeing and mental health’ (26–39, gay/lesbian)
‘Telephone and emails from fertility nurse at the clinic really helped’ (26–39, heterosexual)
‘Clinic does a weekly Q+A online which has been fantastic’ (26–39, heterosexual)
‘A support group was created with regular updates from the clinic and the HFEA website’ (26–39, heterosexual)
‘My clinic advertised fertility yoga sessions via Zoom. Although I haven’t participated, it was good to see that they were trying to get people to use their time to help with their treatment. Doing something will at least make people feel more in control’ (26–39, gay/lesbian)

News articles during the time of clinic closures indicated the harmful impact treatment cancellation was having on the mental health of fertility patients (see Angelides, 2020; Bigg, 2020; Butterly, 2020; Ferguson, 2020; Gurtin, 2020; Kale, 2020; Nargund, 2020). It is thus imperative that lessons are learnt from the positive support mechanisms implemented by some fertility clinics; such support should be adopted across the board and standardised as an integral component of care. Technology can be readily utilised to reach large numbers of patients and, as outlined by respondents, can allow clinics to dispense information, answer questions, offer support and even provide fitness classes, all of which can contribute towards having a positive impact on the mental (and physical) wellbeing of patients.

## Concluding thoughts and recommendations

This article has demonstrated the psychological and sociological implications treatment cancellation has had on female fertility patients in the UK. Increased stress levels due to treatment cancellation has had a detrimental impact on the emotional health and wellbeing of patients, made worse in some cases by the poor support and communication offered by fertility services. Twenty-three percent (*n* = 29) of respondents were not even contacted by their clinics when treatment was cancelled. Instead, they had to actively contact their clinic or managed to retrieve confirmation of their clinic’s closure through social media channels or websites.

With patients experiencing heightened states of isolation and uncertainty, the closure of fertility clinics should be approached with caution to avoid a range of detrimental effects; from poor mental health to permanent childlessness for those in higher age categories. Emerging research is indicating that prolonged periods of lockdown are detrimental to fertility patients and wider society (Alviggi et al., 2020) and it is questionable as to whether subsequent waves of COVID-19 should result in fertility clinic closure again. Indeed, the number of expected births compromised by the lockdown ‘might be as significant as the total number of deaths attributed to the COVID-19 pandemic’ (Alviggi et al., 2020: 2). Furthermore, the subsequent stress resulting from cancelled treatment may have long-lasting effects. Although research has found stress and anxiety to *not* be associated with IVF clinical pregnancy rates (Maroufzadeh et al., 2019), managing stress effectively has been found to help the psychological adjustment of women entering into IVF treatment, reducing the anxiety they may subsequently experience throughout their treatment (Heredia et al., 2020).

As the data in this article illustrate, treatment cancellation produced a range of emotional responses which affected the mental health of fertility patients and impacted their social relationships, particularly within the digital realm. The impact of this is still ongoing with new obstacles now facing those within the TTC community. Fertility clinics in the UK could apply to the HFEA to reopen from mid-May 2020; such news was received positively by the IVF/IUI community, although reopening quickly became a race where some clinics were approved to reopen within days and others within weeks. The resumption of fertility treatment has since looked vastly different, with several social distancing measures being adopted by clinics across the UK. Such measures have included the omission of partners from scans and procedures, the wearing of masks by staff and patients at all times (excluding during sedation), review consultations taking place over the phone or via technology, temperature checks before entering clinics, questionnaires before all appointments to ensure no symptoms of COVID-19 are present, and, in some clinics, mandatory COVID-19 testing at additional costs to patients. If a patient tests positive for COVID-19 at any stage of their treatment, it is cancelled with immediate effect. The financial impact of this is at the discretion of each clinic; for most patients this means financial loss, particularly for those further along in their treatment. Fertility clinics are currently asking that patients shield for the duration of their treatment which, although medically appropriate, adds an additional layer of stress and anxiety to the TTC journey. Further to this, as fertility is not classified as a protected characteristic under the Equality Act 2010, for those in employment, shielding during ART treatment is only at the discretion of their employers, with many workplaces not having IVF policies in place. Shielding, then, is not possible for all patients, particularly those who are key workers and unable to work from home.

It is thus clear that emotional support for fertility patients continues to be needed, as patients now undergo treatment with the fear of the pandemic disrupting their journeys. The positive support from some fertility clinics during clinic closure and the accompanying indeterminate waiting period highlight a range of possibilities for technology to be utilised in constructive and supportive ways. As the world has been forced to become excessively tech-savvy during the global Coronavirus lockdown, fertility clinics should seek to incorporate technology into their wider support services, particularly if the threat of clinic closure looms once more. Regular communication is a vital tool that will positively impact the mental health and wellbeing of fertility patients. Health and welfare services should aim to utilise the benefits of online forums, implementing policies which actively incorporate digital platforms into the care and support services they offer. Emerging research has shown that ‘fertility stakeholders could bolster patient coping by working together to set up transparent processes for COVID-19 eventualities and signposting information and coping resources’ (Boivin et al., 2020: 2565). It is evident that a sense of moral responsibility, beyond the immediate parameters of medical treatment, is needed in fertility care. Several policy recommendations for fertility care have consequently emerged from this paper: (1) fertility clinics should maintain a line of communication with their patients during treatment postponement; (2) fertility clinics should seek feedback from patients during treatment postponement and establish what additional services could be provided during this time; (3) fertility clinics should use the internet more strategically, implementing measures of support which, in the context of the wider pandemic emergency, cannot be implemented in-person; and (4) the HFEA should work towards establishing a guidance for professionals on delivering support during treatment postponement in order to improve coping mechanisms for patients. Emerging research continues to highlight the adverse emotional responses that can arise from treatment postponement (Barra et al., 2020; Boivin et al., 2020; Esposito et al., 2020; Gordon and Balsom, 2020; Trinchant et al., 2020). It is therefore essential that fertility care adopt transformative measures to ensure a more authentic commitment to the wellbeing of patients.

Considering these recommendations, the findings of this study must be seen in light of some limitations. These include an exclusive focus on female fertility patients, predominantly in the 26–39 age category, alongside a COVID-restricted methodology which impeded the possibility of utilising in-person settings. Investigations into the experiences of male fertility patients and partners/support networks of female fertility patients would prove useful, alongside a focus on those in the 40+ age category who are arguably most affected by treatment postponement. Future research on fertility treatment during the COVID-19 crisis should thus seek to adopt an intersectional approach and aim to analyse whether different characteristics lead to improved or worsened states of wellbeing, notably considering gender and age. Men can be overlooked in terms of mental health needs in fertility research and it is important that a rigorous analysis is conducted with masculinity in mind (see Dooley et al., 2014).

It would be further beneficial to investigate global responses to fertility treatment cancellation during the Coronavirus pandemic; the lack of previous research on this topic at the time of data analysis limit the contributions of global perspectives in this paper. Lastly, implications on ‘offstage regions’ being affected during lockdown could offer interesting scope for analysis into stress levels amongst fertility patients, both during treatment cancellation and upon treatment resumption. The psychological, sociological and economic implications of fertility treatment cancellation have been vast, and the subsequent Local Restriction Tier System in the UK, alongside further national lockdowns, have proceeded to cause alarm amongst those undergoing ART treatment. Following the announcement of local restrictions or national lockdowns, online fertility forums and social media pages continue to evidence renewed panic over whether fertility clinics will remain open, demonstrating the additional layer of stress and anxiety COVID-19 has added to fertility treatment. Although the initial cancellation of ART treatment in March 2020 was actioned with good intentions, for a window of time it eradicated the possibility of parenthood for many thousands of couples and individuals unable to conceive naturally, with no government warnings being issued for those still able to pursue natural conception. The negation of reproductive rights must be approached with caution and effective measures should be readily implementable to avoid this happening again should another pandemic threaten the parental hopes of so many in the future.

## Research Data

sj-xlsx-1-hpq-10.1177_1359105321999711 – Research Data for Life on pause: An analysis of UK fertility patients’ coping mechanisms after the cancellation of fertility treatment due to COVID-19 Click here for additional data file. Research Data, sj-xlsx-1-hpq-10.1177_1359105321999711 for Life on pause: An analysis of UK fertility patients’ coping mechanisms after the cancellation of fertility treatment due to COVID-19 by Anna Tippett in Journal of Health Psychology This article is distributed under the terms of the Creative
Commons Attribution 4.0 License ( https://creativecommons.org/licenses/by/4.0/) which
permits any use, reproduction and distribution of the work without
further permission provided the original work is attributed as specified
on the SAGE and Open Access pages ( https://us.sagepub.com/en-us/nam/open-access-at-sage).
